# Polymerases ε and ∂ repair dysfunctional telomeres facilitated by salt

**DOI:** 10.1093/nar/gkw071

**Published:** 2016-02-15

**Authors:** Iglika G. Ivanova, Laura Maringele

**Affiliations:** Institute for Cell and Molecular Biosciences (ICaMB), Newcastle University, Newcastle upon Tyne, NE2 44H, UK

## Abstract

Damaged DNA can be repaired by removal and re-synthesis of up to 30 nucleotides during base or nucleotide excision repair. An important question is what happens when many more nucleotides are removed, resulting in long single-stranded DNA (ssDNA) lesions. Such lesions appear on chromosomes during telomere damage, double strand break repair or after the UV damage of stationary phase cells. Here, we show that long single-stranded lesions, formed at dysfunctional telomeres in budding yeast, are re-synthesized when cells are removed from the telomere-damaging environment. This process requires Pol32, an accessory factor of Polymerase δ. However, re-synthesis takes place even when the telomere-damaging conditions persist, in which case the accessory factors of both polymerases δ and ε are required, and surprisingly, salt. Salt added to the medium facilitates the DNA synthesis, independently of the osmotic stress responses. These results provide unexpected insights into the DNA metabolism and challenge the current view on cellular responses to telomere dysfunction.

## INTRODUCTION

Telomeres are important for preventing DNA loss and DNA damage responses at chromosome ends. When budding yeast telomeres become dysfunctional in the absence of telomerase or of telomere capping proteins, they recruit helicases and nucleases to process the end termini, generating extensive single-stranded DNA (ssDNA) (1). Similarly to yeast, loss of telomere capping leads to increased ssDNA at chromosome ends in mice, chicken and human cells (2–5). In response to ssDNA, cells activate checkpoint pathways to arrest the cell cycle, which provides, among other advantages, time for repair (1). Repair of telomeres appears to involve similar mechanisms to those acting at double strand breaks, for example budding yeast lacking telomerase or the telomere-associated protein Cdc13 uses Rad52-dependent processes to amplify telomeres or subtelomeres. However, repair of telomeres via the Rad52-dependent processes appears to be rarely successful, since less than one in thousand cells emerges from arrest with amplified (sub)telomeres (6–9).

Interestingly, many more cells emerged from arrest if they were exposed to only short periods of telomere dysfunction (10). What happens to the ssDNA lesions formed at telomeres of these cells is not known. One hypothesis is that cells resume proliferation with un-repaired ssDNA lesions. In this case, chromosome ends would considerably shorten following DNA replication, due to the excised strands providing for shorter templates. Short chromosomes lacking telomeres undergo extensive alterations in cells that resume proliferation (11). Another hypothesis is that cells repair the ssDNA lesions, and then resume proliferation. In this case it would be interesting to know which mechanisms were successfully repairing telomeres. Finding out which hypothesis is true is also important for understanding the relationship between telomeres and genome integrity.

Here, we found that cells repaired chromosome ends before resuming proliferation. Repair involved re-synthesis of the double-stranded chromosome ends during cell cycle arrest, which coincided with recruitment of polymerase α, ε and ∂ subunits to damaged (sub)telomeres. We call this process LER (Long-strand Excision Repair). The ability to resume proliferation was independent of Rad52 or factors essential for the error-prone post-replication repair, suggesting that repair was also independent of these processes.

Moreover, we bring evidence of an unexpected connection between the *in vivo* DNA synthesis and salt. Addition of sodium chloride, of other salts, or of sorbitol to the medium facilitated the DNA synthesis by polymerases ε and ∂, and consequently helped cells to resume proliferation, even when the telomere-damaging conditions persisted. Increased salt also facilitated proliferation of cells exposed to alkylating agents or to other DNA damaging conditions, suggesting that salt-facilitated DNA synthesis is not limited to telomeres. In higher organisms, this type of DNA repair could be particularly important for cells undergoing osmotic stress, helping them to maintain viability, proliferation and genomic stability.

## MATERIALS AND METHODS

### Yeast strains, cell culture, serial dilution and cell cycle analysis

All yeast strains were in the W303 background, created either by genetic crossings or by transformation as described previously (12). Gene tagging was performed using the plasmid pFA6a-3HA-natMX6 (13). The *cdc13–1 DPB2-MYC, cdc13–1 POL1-HA* and the BrdU-incorporating *cdc13–1* strains were generated by genetic crossing involving previously described strains: TAY73 *(MAT a ubr1::GAL::UBR1::LEU2 DPB2::6xMYC::kanMX6*) (14), CLY152 (*MATa 3xHA-CDC17 HIS3*) (15) and E3368 (*MAT alpha URA3::GPD–TK (7x)*) (16). Cells were propagated in YPD media (Yeast Extract, Peptone, Dextrose) containing 50 mg/l adenine. Sodium chloride, methyl methanesulfonate (MMS), hydroxyurea (HU) and Phleomycin from Sigma were supplemented to the plates at concentrations indicated in figure legends. Nocodazole was used at 1.5 μg/ml concentrations. For experiments performed on plates, cells grown overnight at 21°C were diluted to about 1.5 × 10^7^cells/ml, followed by 5-fold dilution series, set up in 96-well plates. Small aliquots were transferred to YPD plates using metal prongs. Plates were incubated for 2.5 days at the indicated temperature. To monitor the cell cycle, cells were stained with DAPI and counted by fluorescent microscopy. The following fractions were calculated: cells without buds (in G1 phase), cells with small buds (in S phase), dumbbell shaped cells with one visible nucleus (in G2/M) and dumbbell shaped cells with two visible nuclei distributed between the bud and mother cell (in anaphase/telophase, referred to as late M).

### ssDNA measurements

The real time PCR method QAOS was used to measure ssDNA as previously described (9). Genomic DNA was extracted and equalized to 2 ng/μl at the centromeric locus *PAC2*. Single stranded DNA was measured in the Y’ sub-telomeres (about 600 nt from the chromosome end) and at single gene loci using ssDNA standards as previously described (9). Primers and probes are described in (17) and in the Supplementary data. Experiments were repeated as indicated in the Supplementary Table S1. A representative experiment is shown in the figures. Error bars represent the standard deviation of triplicate measurements from this experiment.

### Chromatin immunoprecipitation (ChIP)

ChIP was performed as previously described (18). Pol1-HA, Pol3-HA and Dpb2-MYC were immuno-precipitated with monoclonal anti-HA (11867423001, Roche) and anti-MYC (Sc-40, Santa Cruz) antibodies. Rap1, Pol2, Dpb11, PCNA and the background were detected with the following antibodies against: Rap1 (sc-6663, Santa Cruz), Pol2 (sc-6753, Santa Cruz), Dpb11 (sc-12007, Santa Cruz) and PCNA (NB500–106, Novus Biological) and goat (cs-2020, Santa Cruz). For each time point, the background (IP with anti-goat antibodies) was subtracted from the immunoprecipitated DNA, and the difference normalized to the input. All these fractions were quantified by real-time PCR (StepOne Plus, Applied Biosystems) using genomic DNA standards. To exclude telomere-unspecific events, the IP at ‘CEN’ (e.g. a centromere-proximal locus, either *PAC2* or *ERG26*) was subtracted (at each time point) from the IP at sub-telomeric and *YER188W* regions. Experiments were repeated as indicated in the Supplementary Table S1. A representative experiment is shown in the figures. Error bars represent the standard deviation of triplicate measurements from this experiment.

### Hog1 immunoprecipitation

To detect Hog1 phosphorylation, proteins were extracted with 10% TCA and resolved on 10% gels. Total Hog1 was detected with a polyclonal anti-Hog1 antibody (sc-6815, Santa Cruz), while phosphorylated Hog1 with a phospho-p38 MAPK (Thr180/Tyr182) antibody (9211S, New England Biolabs), as previously described (19).

### BrdU incorporation

BrdU incorporation was detected by immunoprecipitating DNA fragments with monoclonal anti-BrdU antibody (555627, DB Bioscienses). Cells were grown in the presence of 200 μg/ml 5-bromo-2′-deoxyuridine (BrdU) form Sigma in the dark. Afterward, the BrdU labeled DNA was isolated by phenol-chlorophorm as described (20). The DNA was sonicated to produce fragments of ∼500 bp. The DNA was diluted in FA lysis buffer (50 mM HEPES pH 7.5, 150 mM NaCl, 1 mM EDTA pH 7.6, 1% Triton-X, 0.1% sodium deoxycholate). Each 800 μl sample was incubated with 3 ng BrdU or with goat antibodies (cs-2020, Santa Cruz) and G-Dynabeads (Invitrogen). The input was 80 μl. After overnight incubation at 4°C, the DNA fragments were eluted with Elution buffer (50 mM Tris-HCl pH 7.6, 10 mM EDTA, pH 7.6 and 1% SDS) for 10 min at 65°C. DNA was purified with Qiagen PCR purification kit and enrichment of BrdU at different loci was measured as described for ChIP.

### Telomere blotting

Telomere blotting was performed on Xho1-digested genomic DNA, extracted with phenol-chlorophorm, as previously described (21). The probe was synthesized by PCR using a plasmid containing the TG sequence as a template and labeled with digoxigenin (DIG) using the PCR DIG probe synthesis kit (Roche).

## RESULTS

### DNA polymerases accumulate at chromosome ends during telomere dysfunction

**Figure 1.**
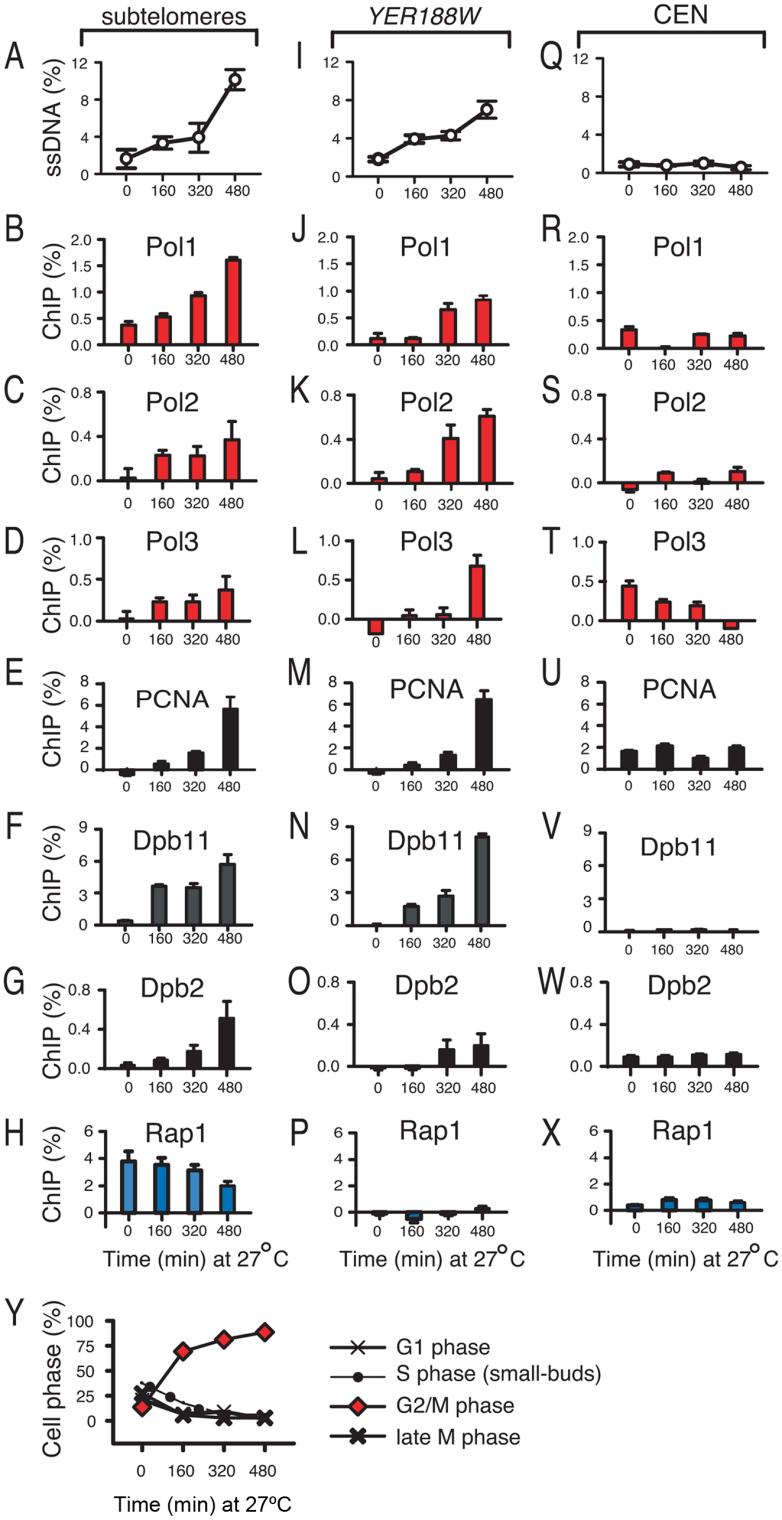
The association of Pol1, Pol2, Pol3, PCNA, Dpb11, Dpb2 and Rap1 with chromosomes during telomere dysfunction. All strains were *cdc13–1*, grown at 21°C, followed by 480 min at 27°C to induce telomere dysfunction. (**A**) Dynamics of ssDNA accumulation in sub-telomeres. (**B–H**) Dynamics of protein association with sub-telomeres. Proteins are indicated above each graph. Error bars are the standard deviation between three measurements. (**I**) Dynamics of ssDNA accumulation at the *YER188W* single gene locus. (**J–P**) As in (B–H), except that the protein association with *YER188W* was analyzed. (**Q**) ssDNA at the centromere-proximal locus *PAC2*. (**R–X**) Protein association with *PAC2*. Pol1 and Pol3 are tagged with HA. Dpb2 is tagged with Myc. Pol2, PCNA, Dpb11 and Rap1 are not tagged. (**Y**) The cell cycle distribution of cells analyzed in A–X.

One of the best-studied models of telomere dysfunction is the telomere uncapping caused by a mutation in the essential protein Cdc13, called *cdc13–1*. This mutation renders cells temperature-sensitive: whereas *cdc13–1* cells proliferate at temperatures below 26°C, they activate checkpoints to arrest proliferation in G2/M at higher temperatures, when increasingly more telomeres and adjacent regions become single-stranded. The dynamics of ssDNA generation by nucleases and the checkpoint responses were previously described (10,17,22). However, little is known whether cells attempt to re-synthesize the excised chromosome ends. To test this possibility, we induced telomere dysfunction by exposing *cdc13–1* cells, previously grown at 21°C, to the restrictive temperature of 27°C. At 21°C, telomeres in *cdc13–1* cells are considered functional. Accordingly, we detected only small amounts of ssDNA at time 0 in sub-telomeres and beyond, e.g. at the *YER188W* locus, situated about 8.5 kb from the right end of chromosome 5 (Figure 1A and I). In contrast, ssDNA accumulated in these regions at the restrictive temperature of 27°C (Figure 1A, I), without reaching as far as the centromere (Figure 1Q).

To determine whether telomere-dysfunctional cells attempted to re-synthesize the excised chromosome ends, we tested by ChIP whether major DNA synthesis factors (Pol1, Pol2, Pol3, PCNA, Dpb11 and Dpb2) were recruited to chromosomes ends. Pol1, Pol2 and Pol3 are the catalytic subunits of DNA polymerases α, ε and δ, respectively. Polymerase α-primase complexes initiate DNA re-synthesis and prime Okazaki fragments during S-phase, whereas polymerases ε and δ elongate these fragments. The PCNA sliding clamp is essential for the processivity of DNA polymerases. Dpb11 and Dpb2 are essential subunits of polymerases ε (23). We found that all these major DNA synthesis factors progressively accumulated in sub-telomeres during telomere dysfunction (Figure 1B–G). Their accumulation was largely proportional to the amount of ssDNA measured in Figure 1A. In contrast, the association of Rap1 with sub-telomeres decreased. Since Rap1 is a transcription factor that binds normal chromosome ends (24), its reduced association is consistent with sub-telomeres becoming single-stranded.

Pol1, Pol2, Pol3, PCNA, Dpb11 and Dpb2 also accumulated beyond sub-telomeres, e.g. at the *YER188W* locus (Figure 1J–O), whereas Rap1 did not (Figure 1P). These factors were also detected closer to centromeres, including at time 0, however their association did not increase with telomere dysfunction, suggesting that it was due to other events (Figure 1R–X). We considered this association to be part of the ‘background’ and subtracted it from the association detected in subtelomeres and at the *YER188W* locus. The recruitment of DNA synthesis factors to chromosome ends took place while the majority of *cdc13–1* cells were arrested in G2/M (Figure 1Y). In summary, DNA polymerases and PCNA were found to accumulate at excised chromosome ends, during the G2/M phase. However, ssDNA also continued to accumulate, suggesting that either DNA synthesis did not take place, or that it was counteracted by excision.

### Chromosome ends are re-synthesized following telomere dysfunction

**Figure 2.**
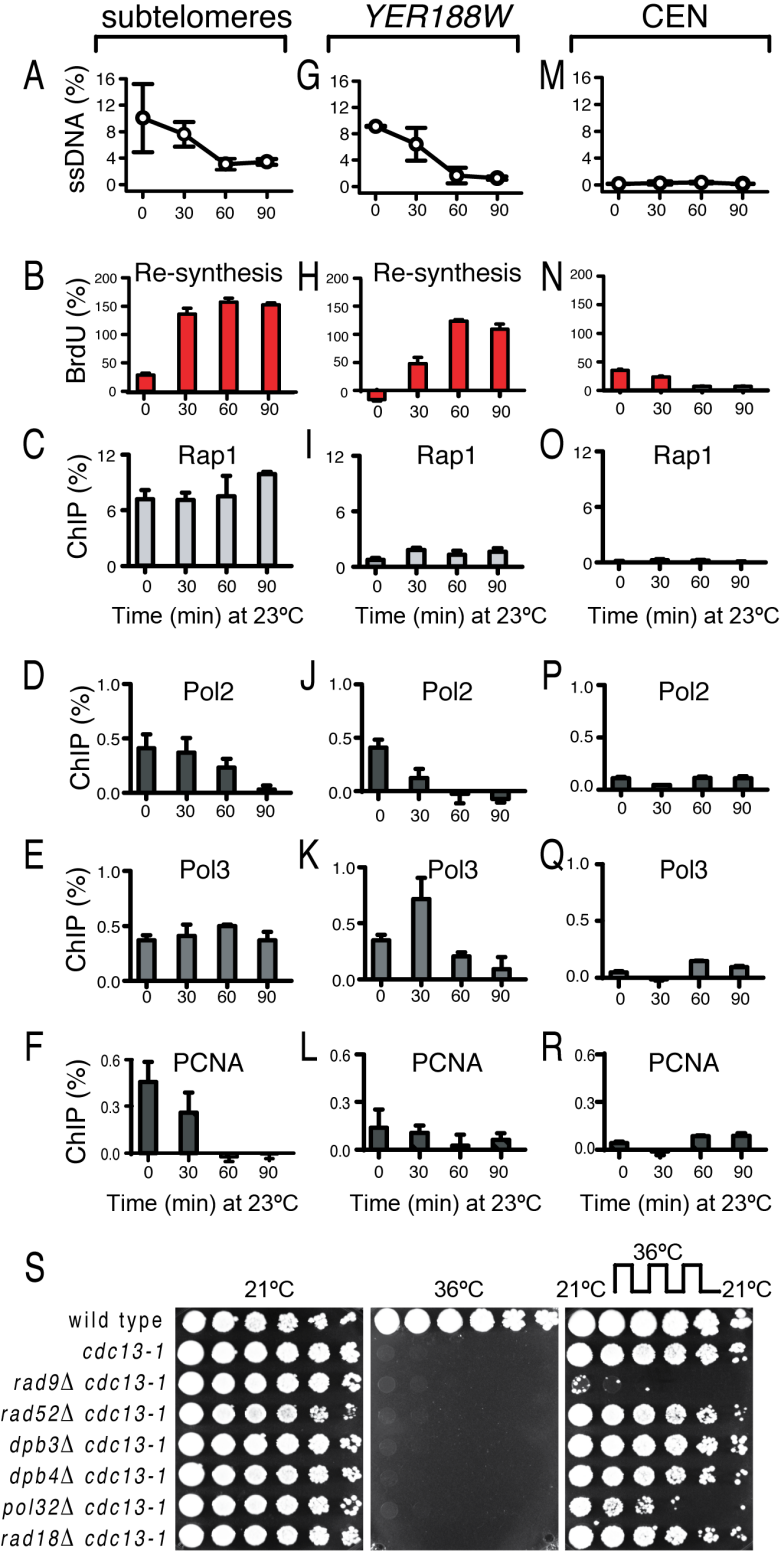
Events taking place during a transient telomere dysfunction. All strains were *cdc13–1*, incubated for 160 min at 36°C to induce telomere uncapping, followed by 90 min at 23°C to allow telomere re-capping. Nocodazole and BrdU were added to the cultures at time 0. (**A**) Dynamics of ssDNA loss in sub-telomeres. (**B**) BrdU incorporation in sub-telomeres, minus incorporation at ‘CEN’ (e.g. a centromere-proximal locus, in this case *ERG26*). (**C–F**) Dynamics of the protein association with sub-telomeres, measured for Rap1 and major DNA synthesis factors (indicated above each graph). ChIP (%) was calculated as the fraction of immunoprecipitated sub-telomeric DNA, minus the fraction precipitated at ‘CEN’ (**G**) Dynamics of ssDNA loss at *YER188W*. (**H**) BrdU incorporation at *YER188W*, minus incorporation at ‘CEN’. (**I–L**) The protein association with *YER188W* was analyzed as in C–F. (**M**) ssDNA at ‘CEN’. (**N**) BrdU incorporation at ‘CEN’. (**O–R**) Association of proteins with ‘CEN’. (**S**) Growth of serial dilution of wild-type (first row) and *cdc13–1* cells with or without additional mutations (indicated on the left of each row) at temperatures indicated above each plate. The plate shown on the right was cycled three times between 4 h at 36°C (to accumulate ssDNA) and 4 h at 21°C (to re-synthesize DNA), followed by incubation at 21°C for another two days.

To determine whether DNA synthesis takes place following telomere dysfunction, we generated *cdc13–1* cells able to incorporate the thymidine analogue BrdU into the newly synthesized DNA (16). These cells were incubated at 36°C for 160 min, to induce telomere dysfunction, and then transferred to 23°C (permissive temperature) for another 90 min, in the presence of BrdU and nocodazole. Nocodazole was used to prevent a cell cycle re-entry and consequently, the BrdU incorporation during DNA replication. We found that following the transfer from 36°C to 23°C, sub-telomeric ssDNA declined (within 60 min) to almost background levels (Figure 2A). At the same time, BrdU accumulated in sub-telomeres, inversely proportional to ssDNA (Figure 2B). Moreover, the Rap1 association increased by 90 min, suggesting that more telomeres became functional and double-stranded (Figure 2C). Furthermore, Pol2 and PCNA were released from sub-telomeres after 60–90 min (Figure 2D–F), whereas Pol3 appeared to persist, possibly due to its role in facilitating the ligation of DNA nicks (25). These data together indicate that (sub)telomeres are rapidly and efficiently re-synthesized after removing cells from a telomere-damaging environment.

Re-synthesis was also detected at the *YER188W* locus. Following the transfer from 36°C to 23°C, the ssDNA at *YER188W* declined to background levels within 60 min (Figure 2G), while increasing levels of BrdU incorporated (Figure 2H). Pol2, Pol3 and PCNA were released from *YER188W* within 60 min (Figure 2J–L). No such events took place closer to centromeres (Figure 2P–R). In conclusion, ssDNA lesions as long as 8 kb are produced and then efficiently re-synthesized outside of the S-phase. We refer to this process as LER, since it involves excision of long strands of DNA, followed by their repair, e.g. DNA re-synthesis.

### Pol32 is important for cell proliferation after a transient telomere dysfunction

The catalytic subunits of DNA polymerases ε and δ are associated with non-essential proteins: Pol2 associates with Dpb3 and Dpb4, whereas Pol3 with Pol32 (26–28). These associations are optimizing the function of DNA polymerases, e.g. Pol32 is essential for the break-induced replication (BIR), a process maintaining telomeres in the absence of telomerase (29). Therefore, we tested whether non-essential components of DNA polymerases and other factors were important for cell proliferation after a transient telomere dysfunction. Serial dilutions of *cdc13–1* cells, with or without other mutations, were spotted onto plates and incubated either at 21°C, at 36°C or cycled three times between 36°C and 21°C (4 h at each temperature) and then incubated at permissive temperature for a few days (Figure 2S). It is clear that growth ceased at 36°C. However, *cdc13–1* cells proliferated well when cycled between 36°C and 21°C, suggesting that the telomere damage accumulating during 4 h at 36°C was being successfully repaired during the next 4 h at 21°C, so that telomeres regained their function. In contrast, repair was too late or too little for *rad9Δ cdc13–1* cells, which did not proliferate, consistent with previous data (30).

We found that many *cdc13–1 pol32Δ* cells failed to grow after being cycled between 36°C and 21°C (Figure 2S), indicating that Pol32 plays an important role when telomeres are regaining their function. In contrast, *dpb3Δ* or *dpb4Δ* mutations did not affect proliferation of *cdc13–1* cells under similar conditions (Figure 2S). Moreover, a *rad52Δ* mutation that abolishes BIR and homologous recombination, or a *rad18Δ* mutation that impairs the function of translesion polymerases (31–33), did not affect proliferation (Figure 2S). In conclusion, Pol32 was important for proliferation of cells undergoing LER during a transient telomere dysfunction. Although Dpb3 and Dpb4 appeared less important, this does not exclude a role for polymerase ε in LER, especially since its catalytic subunit Pol2 (which can act independently of Dpb3 and Dpb4) accumulated at chromosome ends during excision (Figure 1C and K) and declined during re-synthesis (Figure 2D and J). However, we could exclude that BIR, homologous recombination or the translesion synthesis contributed to cell growth following a transient telomere dysfunction, which strongly suggests that these repair activities do not contribute to LER.

### Salt facilitates proliferation of cells with telomere dysfunction

Data presented in Figure 1A indicated that excised chromosome ends were not efficiently repaired when the telomere-damaging conditions persisted, since ssDNA remained high. This could be explained by excision prevailing over repair and/or by repair being inhibited, opening the possibility of screening for factors that could improve repair of telomeres. Therefore, we tested different conditions and found that supplementing the medium with salt made a difference to the growth of telomere dysfunctional cells.

**Figure 3.**
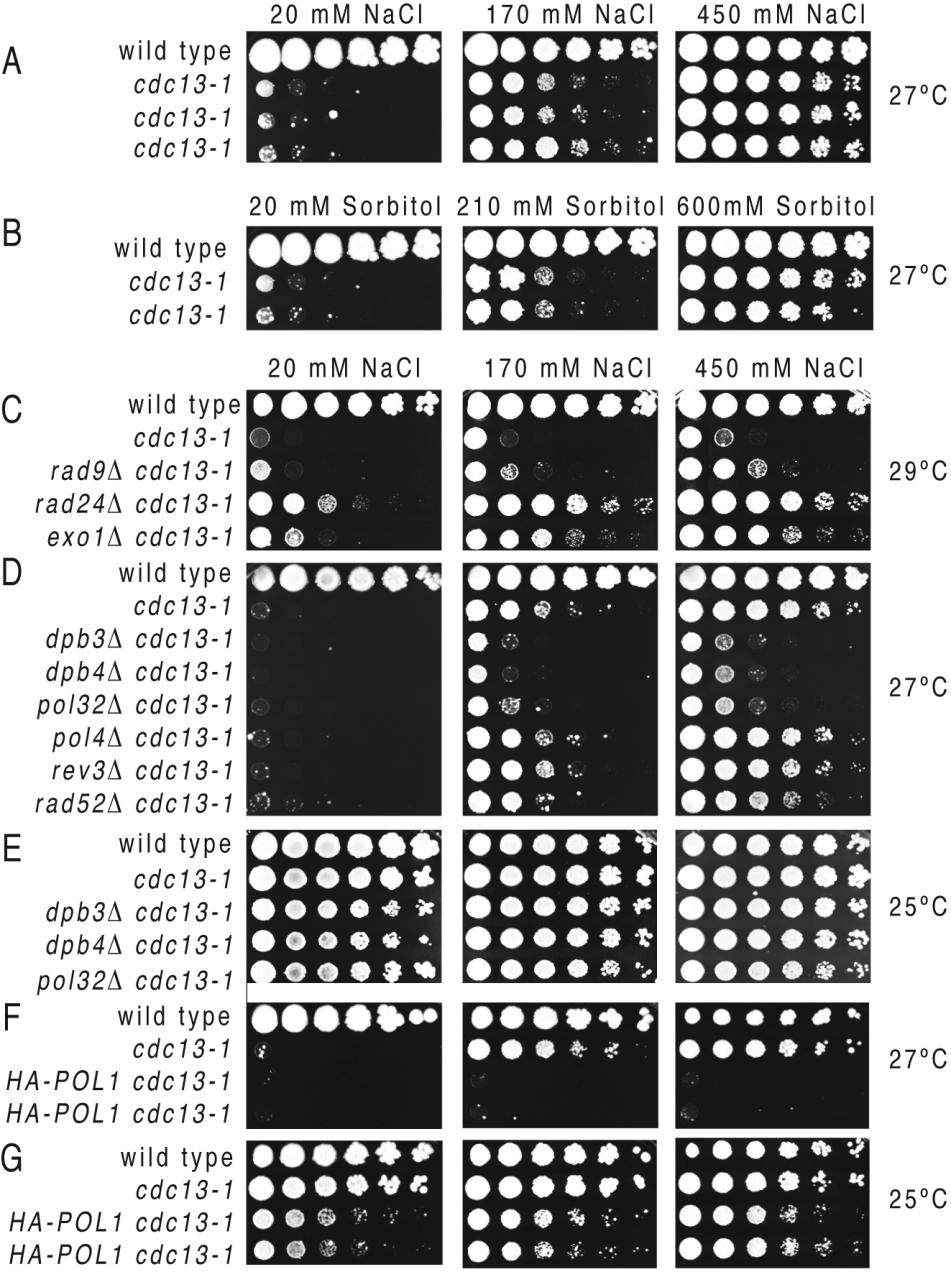
The effect of salt or sorbitol on proliferation of telomere dysfunctional cells. Growth of serial dilution of wild-type (first row of each plate) and *cdc13–1* cells with or without additional mutations (indicated on the left of each row) at temperatures indicated on the far right. (**A, C–G**) The amount of NaCl on each plate is indicated above the plate columns: 20 mM NaCl (left), 170 mM NaCl (middle) and 450 mM (right). (**C**) Serial dilutions of cells incubated at 27°C on YPD plates supplemented with different amounts of sorbitol: 20 mM (left), 210 mM (middle) and 600 mM (right).

A typical yeast YPD medium contains 20 mM NaCl. Several cultures of *cdc13–1* cells were incubated with different concentrations of sodium chloride: 20 mM, 170 mM and 450 mM NaCl. Interestingly, we found that proliferation of *cdc13–1* cells at 27°C was rescued proportional with the concentration of salt (Figure 3A). To determine whether the ability to rescue proliferation of cells with dysfunctional telomeres was specific to NaCl, or more generally caused by an increase in osmotic pressure, we tested the effect of sorbitol at concentrations creating the same osmotic pressure as sodium chloride. We found that sorbitol had similar effects to NaCl in rescuing proliferation of *cdc13–1* cells at 27°C (Figure 3B). Other salts (calcium chloride and magnesium chloride) had similar effects to NaCl or sorbitol (Supplementary Figure S1A). In conclusion, salt and/or osmotic pressure facilitate the proliferation of cells undergoing LER.

### Pol32, Dpb3, Dpb4 and Pol1 are required for the salt-effect at telomeres

Deletion of genes required for telomere excision like *EXO1*, or deletion of checkpoint genes essential for cell cycle arrest in response to telomere dysfunction, like *RAD9* and *RAD24*, are also known to rescue proliferation of *cdc13–1* cells, up to 28°C (17). Therefore, we tested whether the effect of salt required these genes. We found that this was not the case, because increased salt also rescued proliferation of *cdc13–1* cells with *exo1Δ, rad9Δ* or *rad24Δ* mutations at 29°C, a temperature which is restrictive for growth of these mutants (Figure 3C). Therefore, salt has synergistic effects with that of checkpoints or Exo1 mutations, which suppress the cell cycle arrest and the telomere excision, respectively, suggesting that salt is promoting cell proliferation through a different mechanism.

Salt may facilitate repair of telomeres by facilitating activities like BIR/telomere recombination, or perhaps the ssDNA gap repair by translesion polymerases, like polymerase zeta. We found that salt also rescued proliferation of *cdc13–1* cells lacking *POL4* or *REV3*, encoding the catalytic subunits of DNA polymerase IV and polymerase zeta, respectively, suggesting that these polymerases are not required for the salt effect (Figure 3D). Similarly, salt rescued proliferation of *cdc13–1 rad52Δ* cells, indicating that its effect was independent upon BIR or upon homologous recombination.

Salt may facilitate the re-synthesis component of LER. Importantly, salt had little effect on proliferation of *cdc13–1* cells with a *pol32Δ, dpb3Δ* or *dpb4Δ* mutations (Figure 3D). The lack of rescue was not due to some toxic effect of salt, since *cdc13–1* cells with *pol32Δ, dpb3Δ, dpb4Δ* proliferated well at 25°C under high salt conditions (Figure 3E). The *pol32Δ, dpb3Δ, dpb4Δ* single mutants also proliferated well on high salt (Supplementary Figure S1C). Moreover, salt had little effect in rescuing the proliferation of *cdc13–1* cells with a *HA-POL1* construct (Figure 3F and G). Tagging *POL1* (with HA) may limit its function during telomere uncapping, since *HA-POL1 cdc13-1* were more temperature sensitive that *cdc13–1* single mutants at 25°C (Figure 3G), whereas they grew well at 21–24°C, when telomeres in *cdc13–1* are considered functional (Supplementary Figure S1B). The limited function did not prevent *HA-POL1* from accumulating at chromosome ends during telomere uncapping (Figure 1B). Other constructs used in this study did not affect the cellular proliferation (Supplementary Figure S1B). In conclusion, the effect of salt on enhancing proliferation of telomere-dysfunctional cells requires a fully functional Pol1, as well as the accessory subunits of polymerases ε and δ, whereas it is independent upon BIR, telomere recombination and the translesion DNA synthesis.

### Salt facilitates the DNA synthesis

**Figure 4.**
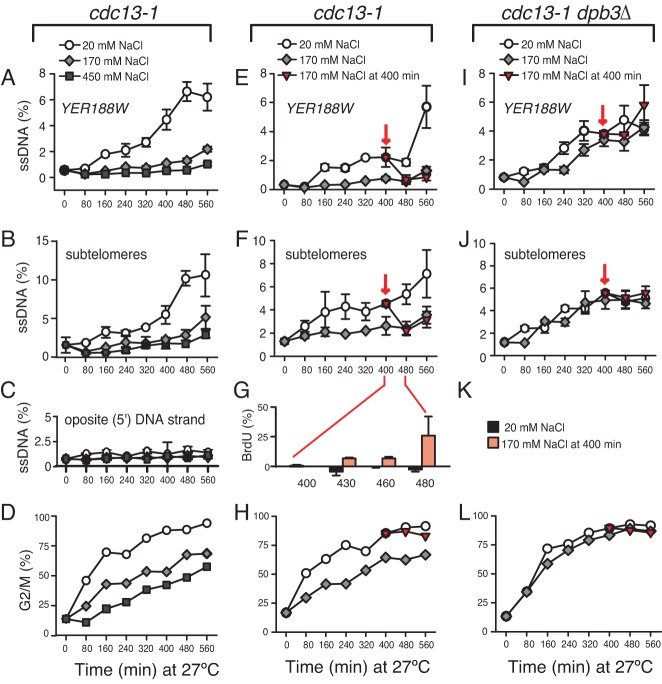
The effect of salt on DNA synthesis. Strains were *cdc13–1* or *cdc13–1 dpb3Δ*, grown overnight at 21°C, followed by 560 min incubation at 27°C in YPD medium with different concentration of salt. (**A–D**) *Cdc13–1* cells were incubated at 27°C with 20 mM NaCl (white circles), 170 mM NaCl (grey rombi) or 450 mM NaCl (dark sqares); (**A**) Dynamics of ssDNA accumulation at *YER188W* and (**B**) in sub-telomeres; (**C**) 5′-ended ssDNA measured in sub-telomeres; (**D**) The fraction of cells accumulating in the G2/M phase; (**E–H**) *Cdc13–1* cells were incubated at 27°C with 20 mM NaCl (white circles), 170 mM NaCl (grey rombi). After 400 min, half of the 20 mM NaCl culture was supplemented with salt to 170 mM NaCl (dark triangles) and incubated further; (**E**) Dynamics of ssDNA accumulation/loss at *YER188W* and (**F**) in sub-telomeres; (**C**) BrdU incorporation in sub-telomeres of cells incubated with 20 mM NaCl (black columns) and with 170 mM supplemented at 400 min (light grey columns); (**H**) The fraction of cells accumulating in the G2/M phase; (**I–J, L**) *Cdc13–1 dpb3Δ* cells were incubated as described for (E–H); (**I**) Dynamics of ssDNA accumulation at *YER188W* and (**J**) in sub-telomeres; (**K**) Legend for G; (**L**) The fraction of cells accumulating in G2/M.

Salt may facilitate cell proliferation by inhibiting the accumulation of ssDNA. To test this hypothesis, liquid cultures of *cdc13–1* cells were incubated at 27°C with different concentrations of sodium chloride and the dynamics of ssDNA assessed by QAOS (9). We found that high levels of ssDNA (e.g. of the 3′-ended strand), about 7–10%, accumulated in subtelomeres and beyond (at *YER188W*) in *cdc13–1* cells incubated with 20 mM NaCl (Figure 4A and B). In contrast, 2- to 3-fold less ssDNA was detected in these regions, when cells were incubated with 170 or 450 mM NaCl (Figure 4A and B). No significant ssDNA was detected when quantifying the opposite strand (5′-ended), at the same locus, irrespective of salt (Figure 4C). Moreover, the fraction of G2/M arrested cells significantly lowered with added salt (Figure 4D). Therefore, salt prevents the accumulation of ssDNA, which explains why telomere dysfunctional cells proliferate better with increased salt concentrations.

To prevent the ssDNA accumulation, salt may somehow increase the protection at chromosome ends, or it may facilitate the DNA re-synthesis. To distinguish between these hypotheses, we examined the effect of salt on previously accumulated ssDNA. If salt works through preventing ssDNA accumulation, it should not affect the amount of ssDNA already accumulated in cells. In contrast, if salt works through facilitating DNA synthesis, the amount of ssDNA should decrease following the addition of salt to the medium. For these experiments, *cdc13–1* cells were incubated at 27°C in conventional YPD (20 mM NaCl). After 400 min, half of the culture was supplemented with BrdU and with salt to the final concentration of 170 mM. The other half was treated with BrdU only. All cells were incubated at 27°C for an additional 160 min. Another culture of *cdc13–1* cells was incubated with 170 mM NaCl at 27°C from the beginning to the end of the experiment (e.g. for 560 min).

We found that ssDNA measured at *YER188W* (Figure 4E) and in sub-telomeres (Figure 4F) decreased by 2-fold within 80 min following the addition of salt, to values indistinguishable from those in cells incubated with 170 mM NaCl from the beginning of the experiment. To determine whether this ssDNA decrease was due to DNA synthesis, we monitored the BrdU incorporation. No significant BrdU incorporation (e.g. above the background level measured close to centromeres) was detected in sub-telomeres of cells incubated with 20 mM NaCl. In contrast, increasing amounts of BrdU were incorporated between 430–480 min, e.g. 30–50 min after addition of salt (Figure 4G). The majority of cells remained arrested in G2/M during that time, (Figure 4H), indicating that BrdU incorporation took place outside of the S-phase. Taken together, these data indicate that salt facilitates LER under persisting telomere damaging conditions.

### Dpb3 is important for the salt facilitated LER

Figure 3D shows that the effect of salt in facilitating proliferation of cells under persistent telomere damaging conditions requires the accessory subunits of polymerases ε and δ. This strongly suggests that these subunits (Dpb3, Dpb4 and Pol32) are important for the repair component of LER on salt. To test this hypothesis, *cdc13–1 dpb3Δ* cells were incubated at 27°C in the presence of 20 or 170 mM NaCl. After 400 min, half of the 20 mM NaCl culture was supplemented with salt to the final concentration of 170 mM NaCl.

Whereas addition of salt triggered a decrease in ssDNA levels in *cdc13–1* cells (Figure 4E and F), addition of salt to *cdc13–1 dpb3Δ* cultures had little effect (Figure 4I and J). Indeed, ssDNA continued to accumulate in *cdc13–1 dpb3Δ* cells, irrespective of the salt concentration, even when salt was supplemented from the beginning (e.g. at time 0). Consistent with persisting ssDNA, *cdc13–1 dpb3Δ* cells arrested in G2/M, irrespective of the amount of salt (Figure 4L). These data indicate that Dpb3 (and most likely also Dpb4 and Pol32) are important for the salt-facilitated telomere synthesis.

### LER is independent upon major osmotic stress factors: Hog1, Msn2 and Msn4

**Figure 5.**
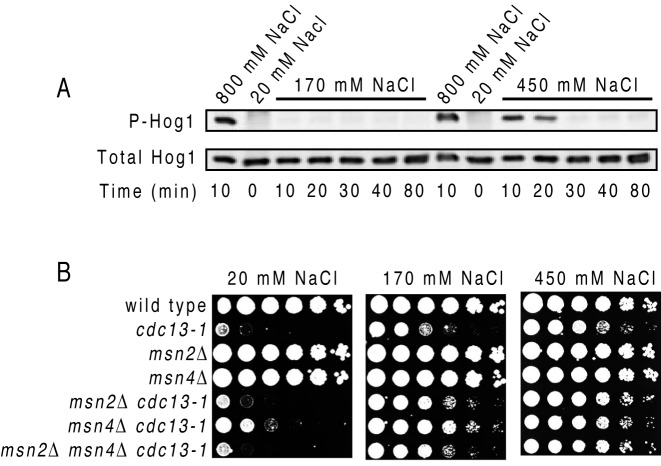
The effect of salt on Hog1 phosphorylation and proliferation of cells lacking Msn2, Msn4. (**A**) The effect of different concentrations of salt (indicated above the picture) on phosphorylation of Hog1, detected with a phospho-specific antibody (top lane). The total amount of Hog1 was also detected (bottom lane). (**B**) Growth of serial dilution of wild-type and strains with mutations (indicated at the left of each row) on plates with different concentrations of salt (indicated above the plates), incubated for 3 days at 27°C.

The salt-facilitated DNA synthesis could be an osmotic stress response. Osmotic stress results in cell shrinkage and therefore it activates various pathways cooperating to restore the normal cell size, e.g. by producing glycerol inside the cell and affecting protein synthesis (e.g. mRNA stability). One major osmotic stress pathway is the Hog1 pathway, activated by phosphorylation of Hog1 (34). Therefore, we tested whether 170, 450 or 800 mM NaCl triggered Hog1 phoshorylation in *cdc13–1* cells, using a previously validated phospho-p38 antibody (19). Whereas Hog1-phoshorylation was clearly detected after 10 min in 400–800 mM NaCl, no such signal was detected in 170 mM NaCl, even after 80 min (Figure 5A).

In conclusion, Hog1 was not activated by salt concentrations that facilitated DNA synthesis and cell proliferation (e.g. by 170 mM NaCl), and therefore these events are most likely Hog1-independent. This conclusion was supported by further experiments, testing whether Msn2 and Msn4 transcription factors, which act downstream of Hog1 in the osmotic stress responses (34), were required for the salt-facilitated proliferation of *cdc13–1* at non-permissive temperatures. We found that neither Msn2 nor Msn4 were required, since salt also rescued proliferation of *cdc13–1* with single *msn2Δ, msn4Δ* or with double *msn2Δ msn4Δ* mutations (Figure 5B). We conclude that salt facilitates LER independently upon major osmotic stress response factors.

### Salt rescues proliferation of *yku70Δ, cdc9–1*, MMS and Phleomycin-treated cells

**Figure 6.**
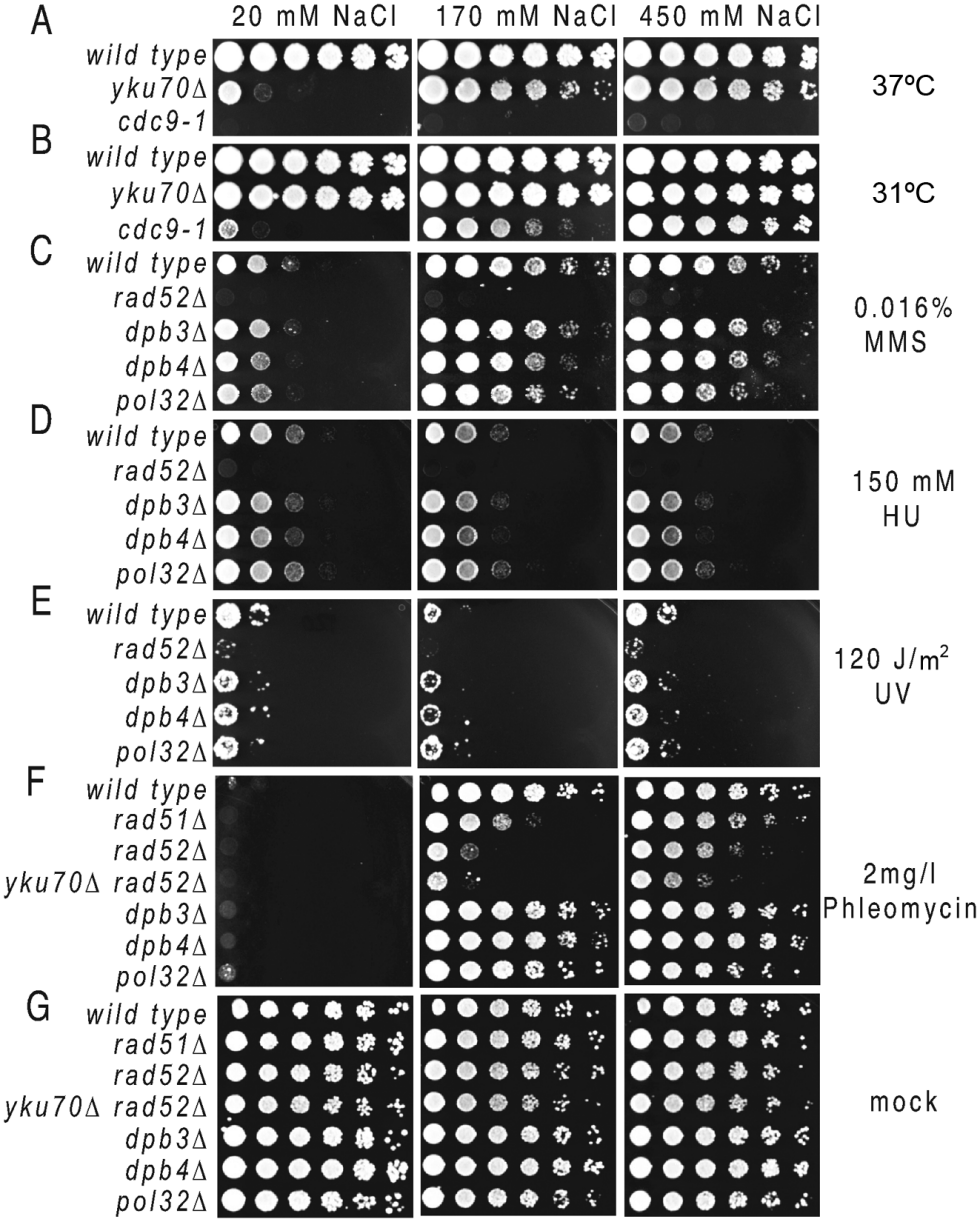
The salt effect on proliferation of *yku70Δ, cdc9–1*, MMS, HU, UV and Phleomycin-treated cells. Growth of serial dilution of wild-type and strains with a deletion mutation (indicated at the left of each row) on plates with different concentrations of salt (as indicated above the plates) incubated at restrictive temperatures or treated with MMS, HU, UV or Phleomycin. (**A**) Plates were incubated at 37°C; (**B**) at 31°C; (**C**) Plates contained 0.016% MMS; (**D**) 150 mM HU or (**F**) 2 g/l Phleomycin; (**E**) Cells were irradiated with UV at 120 J/m^2^. (**G**) Cells were mock treated. Unless otherwise specified, plates were incubated at 23°C (F–G) or 25°C (C–E). Other concentrations and mock treated cells are presented in the Supplementary Figure S2.

We have shown that salt facilitates the DNA synthesis component of LER during a persistent telomere dysfunction caused by exposing *cdc13–1* cells to restrictive temperature. However, DNA synthesis is also required during many other types of DNA damage. Therefore, we tested whether salt was also able to rescue proliferation of cells undergoing the followings: (i) *yku70Δ*-dependent telomere uncapping; (ii) *cdc9–1*-dependent DNA replication defect; (iii) DNA alkylation caused by MMS, (iv) inhibited DNA synthesis caused by hydroxyurea (HU); (v) pyrimidine dimers caused by UV and (vi) DNA damage caused by Phleomycin (Figure 6 and Supplementary Figure S2).

Cells with *yku70Δ* mutations in the telomere capping and double-strand break (DSB) repair Yku70/Yku80 complex are temperature sensitive above 36°C (35–37), due to the accumulation of sub-telomeric ssDNA generated by the Exo1 nuclease (22). We found that salt rescued proliferation of *yku70Δ* cells in a dose dependent manner, similarly to what we observed in *cdc13–1* cells (Figure 6A). The fact that salt also rescued a gene deletion mutant makes the hypothesis that salt works through improving the function of mutant proteins like *cdc13–1*, less probable.

Salt also rescued proliferation of *cdc9–1* cells at 31°C, but not at 37°C (Figure 6A and B). These cells have a point mutation in *CDC9* encoding the DNA ligase I, required to join the Okazaki fragments following DNA synthesis. The fact that *cdc9–1* cells proliferate better with increased salt concentration may suggest that they undergo a salt-facilitated LER at some of the DNA nicks caused by defective fragment ligation.

Salt also rescued proliferation of wild-type cells exposed to MMS (Figure 6C and Supplementary Figure S2). MMS methylates adenine and guanine (38). Repair of methylated adenine by the base excision repair (BER) machinery generates short ssDNA lesions (39). Moreover, salt rescued growth of *dpb3Δ, dpb4Δ* and *pol32Δ* mutants on MMS, but not that of *rad52Δ* mutants. These data suggest that salt also facilitates BER, however Dbp3, Dpb4 and Pol32 are less relevant for this process as they are for the salt-facilitated LER.

HU blocks DNA synthesis by decreasing the availability of dNTPs. In consequence, cells treated with HU arrest in the S-phase (40). We found that salt did not rescue proliferation of wild-type cells incubated with 120–150 mM HU (and therefore unable to synthesize DNA). In fact, 450 mM NaCl appeared to inhibit proliferation of HU-treated cells (Figure 6D and Supplementary Figure S2). This result supports the conclusion that salt facilitates the DNA synthesis, since blocking DNA synthesis removes the effect of salt on cell proliferation. Another type of DNA damage apparently unaffected by salt was caused by UV irradiation (Figure 6E). One explanation for the lack of effect could be that the longest ssDNA lesions generated during the UV damage repair are much shorter than in LER, about 2–15 nucleotides (41).

In contrast, salt rescued proliferation of cells treated with Phleomycin, which produces single and double-stranded breaks in DNA. It is clear from Figure 6F that defects in DSB repair (progressing in severity from *rad51Δ*, over *rad52Δ* to *rad52Δyku70Δ* cells) correspond to similarly incremental growth defects on 170 mM NaCl. However, 450 mM NaCl partially rescued proliferation of these mutants, suggesting that high salt may interfere with the Phleomycin absorption (Figure 6F). This effect could be overcome by increasing the concentration of Phleomycin, which stops *rad52Δ* cells from growing on high salt (Supplementary Figure S2D). In contrast, salt rescues proliferation of wild-type, *dpb3Δ, dpb4Δ* and *pol32Δ* cells (Figure 6F). These data suggest that salt may facilitate the DNA synthesis following DSBs, yet again the non-essential subunits of polymerases ε and ∂ had little effect.

In conclusion, salt facilitates many other processes in addition to LER. All investigated processes facilitated by salt require DNA synthesis and yet, with the exception of LER, the salt effect is independent of Dpb3, Dpb4 and Pol32. This difference could be caused by a difference in the length of the ssDNA lesions requiring synthesis, with the non-essential subunits becoming important when the lesions are longer, as in LER.

### High salt helps to stabilize the telomere length under stress

One important question is whether or not salt supplements are affecting the telomere length. Interestingly, a previous study has shown that high concentration of salt did not affect the length of wild-type yeast cells (42). However, little is known about the effect of salt on cells undergoing telomere damage. To address this question, we incubated *cdc13–1* cells at restrictive temperature (27°C) for several days, on different concentrations of salt, and analyzed the daily telomere length by Southern blotting (Supplementary Figure S3). We found that the telomere length decreased with time when cells were incubated in medium with 20 mM NaCl or with 170 mM NaCl. This suggests that some of the ssDNA becomes degraded with time. In contrast, the telomere length was maintained at wild-type levels in medium with 450 mM NaCl, suggesting that the ssDNA was efficiently re-synthesized. Moreover, high salt appeared to inhibit the apparition of recombination dependent survivors (indicated by an arrow in Supplementary Figure S3). In conclusion, high salt helps to stabilize the telomere length of cells maintained for several days under telomere-dysfunction conditions.

## DISCUSSION

Telomeres are exposed to many cellular and extracellular factors that could damage them, leading to different issues including cancer, aging and loss of organ function. The prevailing view is that dysfunctional telomeres are most often recognized and processed as DSBs by homologous recombination (HR) or non-homologous end joining (NHEJ) repair pathway. However, there are significant differences in the DNA damage responses to telomeres versus DSBs (43). In this study, we used budding yeast to uncover a novel cellular response to dysfunctional telomeres, response we call LER. The name LER was chosen to be similar to BER or NER (base or nucleotide excision repair) to emphasize that cells often use the same principles, albeit different mechanisms, to deal with modified DNA: excision, followed by re-synthesis. We show that following the removal of *cdc13–1* cells from a telomere-damaging environment, telomeres are successfully repaired and the majority of cells resume proliferation. Interestingly, we found that telomeres of *cdc13–1* can be repaired even when the telomere damaging conditions persist, however, in this case repair requires a supplement of salt.

**Figure 7.**
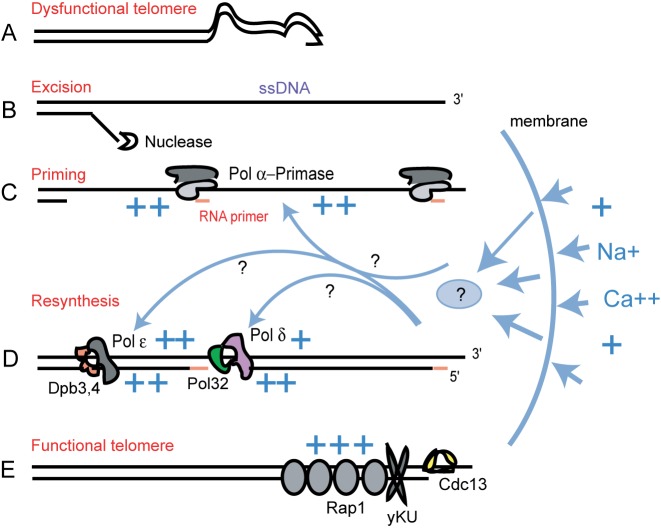
A model for LER and the salt effect at telomeres. The cartoon depicts different stages of LER: (**A**) Telomere dysfunction, e.g. caused by entanglements followed by loss of telomere-associated proteins; (**B**) Excision of the 3′–5′ strand by nucleases and generation of ssDNA; (**C**) Priming by Pol α-Primase complexes; (**D**) Re-synthesis of the ssDNA to dsDNA by Polε and ∂; (**E**) Telomeres regain their structure and function. Salt added to the medium and/or intracellular cations released in response to increased osmotic pressure penetrate the nuclear membrane to facilitate the events described in (**C**) and (**D**), by activating DNA polymerases and/or affecting the DNA substrate. In the absence of extra salt, telomere(s) can still regain their function when the entanglement has been resolved during the B–D stages, or when cells are removed from the telomere-damaging environment.

To explain how LER works, we propose the following model (Figure 7). Dysfunctional telomeres are processed by helicases and nucleases, generating long ssDNA lesions at chromosome ends, which trigger a cell cycle arrest, as described in Figure 1A and consistent with previous data (1). This is the excision phase of LER (Figure 7B). During this phase, DNA Pol α, ε, δ and other relevant factors associate with chromosome ends (Figure 7C). If cells are removed from the telomere-damaging environment, or if their medium is supplemented with salt, re-synthesis of DNA takes place (Figure 7D). The newly resynthesized telomeres recruit the normal telomere-associated factors (Figure 7E) and cells resume proliferation. This is the re-synthesis phase of LER.

LER is independent upon Rad52 and Rad18 (Figure 2S), and therefore independent of other DNA repair processes involving DNA synthesis, e.g. the BIR/homologous recombination and the post-replication repair of ssDNA gaps by translesion polymerase activity. LER is less costly, in terms of outcome, than other repair pathways at telomeres, e.g. BIR and NHEJ. This is because unlike LER, both BIR and NHEJ would change the genome irreversibly; BIR by massively amplifying (sub)telomeres, NHEJ by inducing breakage-fusion-bridge cycles and genomic instability.

We suggest that LER is an evolutionary selected response to transient telomere dysfunction events which may occur often in the wild-type cells, e.g. when proteins like Cdc13 cannot bind to their telomeric substrate, because it is either occupied, entangled or modified beyond recognition (Figure 7A). We propose that nucleases excise one strand of telomeric DNA in order to remove the obstacle, entanglement or modification. DNA re-synthesis then restores a functional telomere and cells resume proliferation. Paradoxically, LER may also eliminate cells from the proliferation pool when functional telomeres cannot be restored, e.g. when telomeres are short and telomerase activity insufficient. This is because a previous study showed that Dpb3 was required for cells to remain arrested (e.g. senescent) in response to telomere attrition (44). An alternation between excision and re-synthesis of telomeres was postulated, and proposed to be important for maintaining senescence, thus preventing chromosomal instability.

In *cdc13–1* cells maintained at restrictive temperature, ssDNA would continue to accumulate. However, when salt is added to the medium, telomeres are repaired and cells resume proliferation, even if the telomere damaging conditions (e.g. restrictive temperature) persisted. Salt triggers DNA re-synthesis and restores the cell proliferation independently of Rad52 or polymerases IV and zeta (Figure 3D). In contrast, the salt effect requires a fully functional Pol1 and also the subunits associated with Pol ε and δ: Dpb3, Dpb4 and Pol32. For example, if cells lack Dpb3, telomeres are not being repaired when salt is added to the medium. These strongly suggest that repair of telomeres maintained in a telomere-damaging environment requires an enhanced activity of Pol ε and δ, achieved by their association with the non-essential subunits.

Importantly, the salt effect is not restricted to telomeres. Although salt did not rescue cells treated with UV, it rescued the proliferation of other DNA damaged cells, e.g. *cdc9–1* cells exposed to restrictive temperature and wild-type cells exposed to MMS and Phleomycin. These cells have nicks in DNA, which may be processed by nucleases into longer ssDNA lesions. Perhaps salt facilitates the DNA re-synthesis after Phleomycin and MMS treatment, similarly to its effect in *cdc13–1* cells (Figure 4). Whether or not the non-essential subunits of polymerases ε and ∂ play a role may depend upon the length of the ssDNA lesions.

To explain how salt facilitates DNA synthesis, we propose several plausible hypotheses. One is that intracellular cations like Ca^2+^ and Mg^2+^, released in response to increased osmotic pressure, may facilitate the activity of DNA polymerases directly (similarly to the effect of cations in polymerase chain reactions) or indirectly, by facilitating their association with other factors. Another hypothesis is based on a study showing that cations are part of the normal chromosome structure (45). We propose that cations may help to stabilize ssDNA lesions, thus facilitating re-synthesis of double-stranded DNA (dsDNA) (Figure 7C and D). However, salt in excess induces DSBs, for example in kidney cells (46–48). To explain the DSB formation in kidney cells, we suggest that cations in excess could lead to DNA configurations that attract enzymes that generate nicks and ssDNA, which may in turn convert to DSBs. To prevent the toxic DSB formation, we hypothesize that cells have evolved to respond to increased concentrations of salt by activating the DNA synthesis machinery. Thus, the majority of ssDNA gaps would be successfully repaired, including those produced by other mechanisms (e.g. telomere uncapping, MMS, Phleomycin) before they could convert to DSBs. To summarize this hypothesis, salt has two related effects: one is to induce formation of ssDNA gaps, the other one is to induce repair of these (and other) ssDNA gaps. The overall outcome is positive (e.g. prevention of DSBs formation and DNA damage responses), except when ssDNA formation exceeds the repair capacity. If true, these effects most likely play important roles in survival of cells under high salt conditions.

High osmotic pressure also occurs in lymphoid organs, liver and plasma cells of patients with diabetes mellitus and inflammatory bowel disease (49). Perhaps the salt-facilitated DNA synthesis helps these cells to avoid some of the toxic effects of increased osmotic pressure. Further investigations will be required to understand the complex interactions between salt/osmotic pressure and DNA metabolism.

## Supplementary Material

SUPPLEMENTARY DATA
