# Supplementary material for: Polymerases ε and ∂ repair dysfunctional telomeres facilitated by salt
Source: Nucleic Acids Res. 2016 Feb 15;44(8):3728–38. doi: 10.1093/nar/gkw071 (PMC4856982; doi:10.1093/nar/gkw071)
Supplement: SUPPLEMENTARY DATA [file supp_44_8_3728__index.html]

Polymerases ε and ∂ repair dysfunctional telomeres facilitated by salt — Polymerases ε and ∂ repair dysfunctional telomeres facilitated by salt — SUPPLEMENTARY DATA 

# Polymerases ε and ∂ repair dysfunctional telomeres facilitated by salt

## SUPPLEMENTARY DATA

- SUPPLEMENTARY DATA
