# Supplementary material for: Polymerases ε and ∂ repair dysfunctional telomeres facilitated by salt
Source: Nucleic Acids Res. 2016 Feb 15;44(8):3728–38. doi: 10.1093/nar/gkw071 (PMC4856982; doi:10.1093/nar/gkw071)
Supplement: SUPPLEMENTARY DATA [file supp_gkw071_nar-02559-d-2015-File009.pdf]

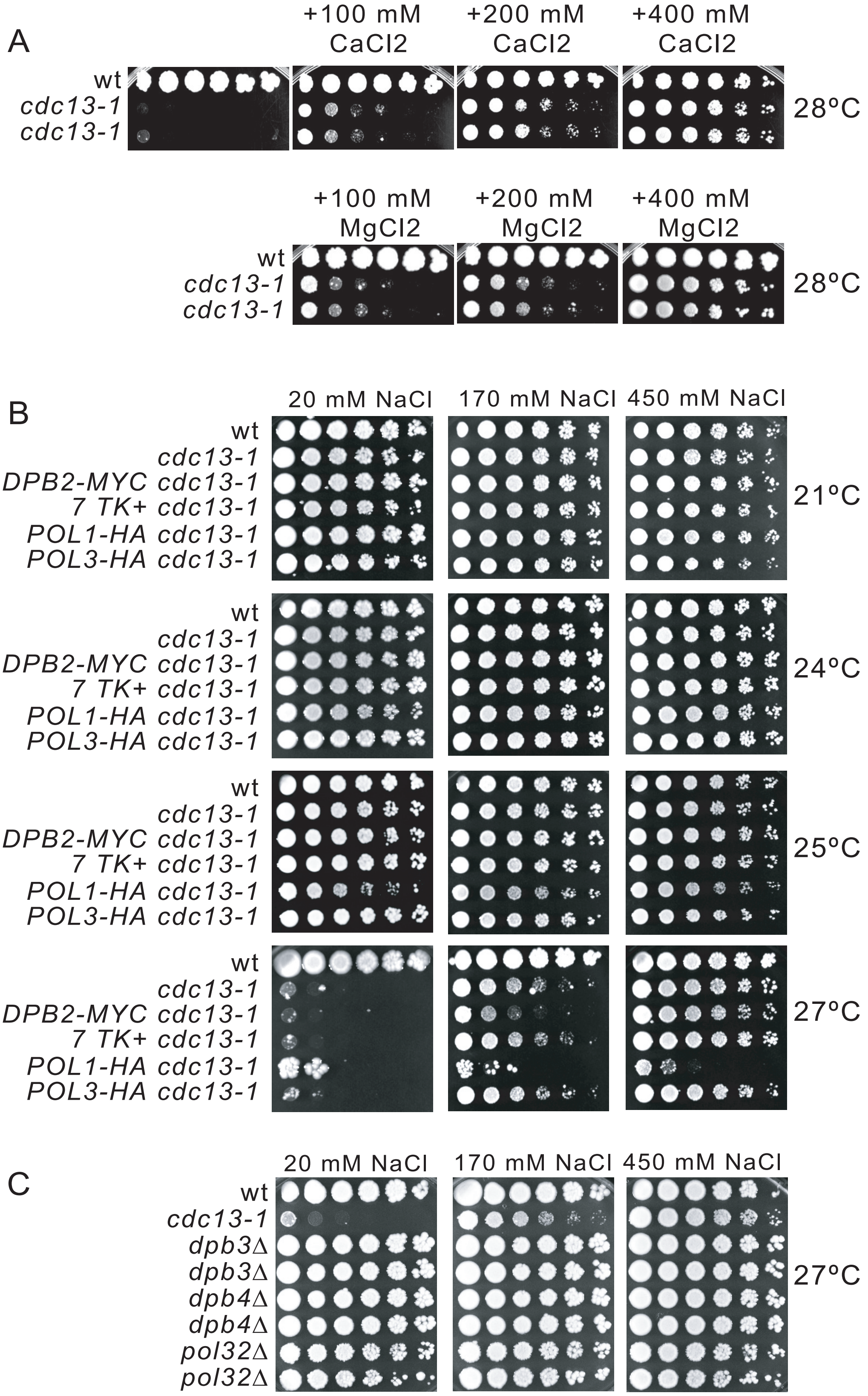

Supplementary Figure 1

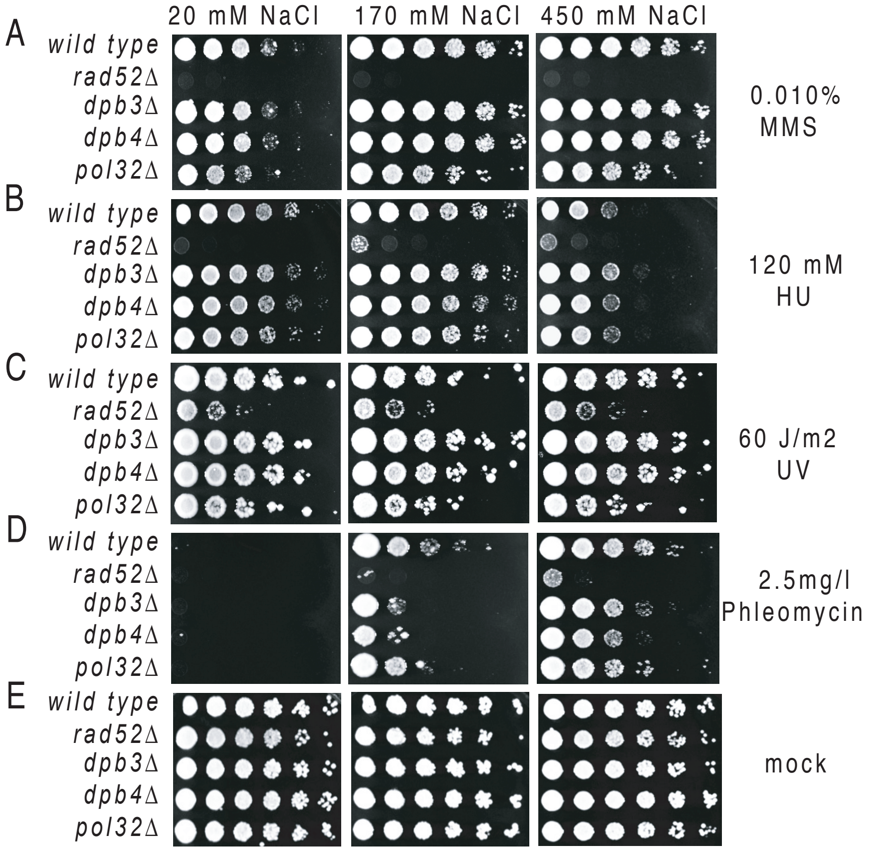

Supplementary Figure 2

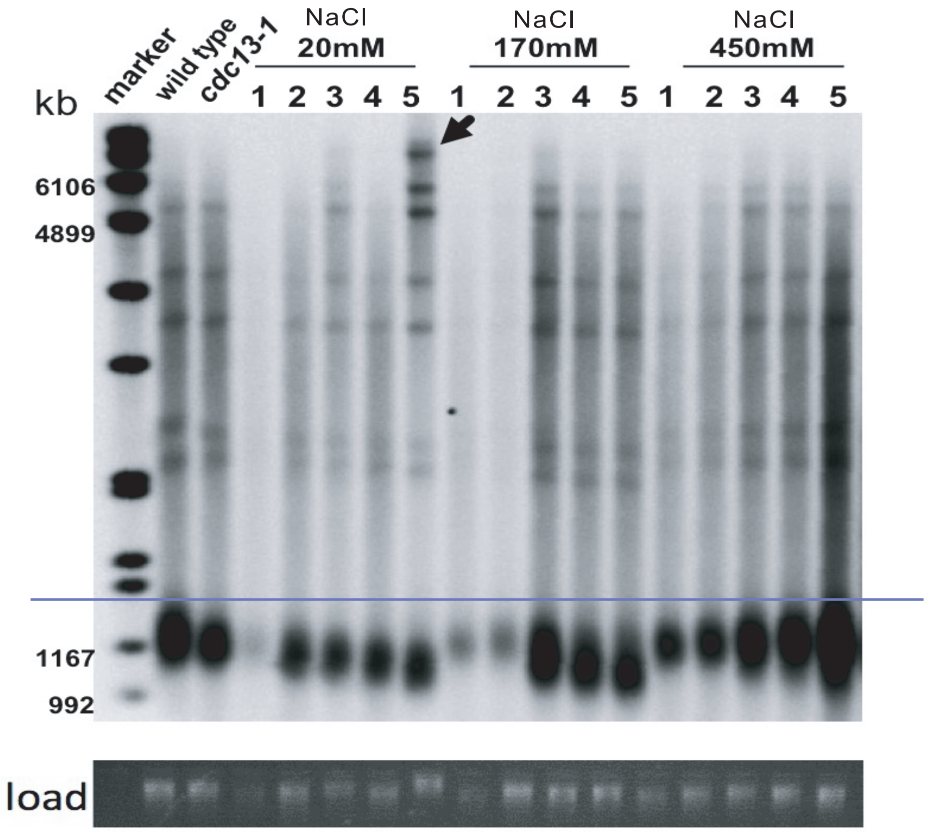

Supplementary Figure 3

Supplementary information

Supplementary Figure 1: The effect of salt on proliferation of different mutants. Serial dilutions of cells with relevant mutations were incubated on YPD plates supplemented with different salts (indicated above plate columns) at temperatures indicated on the right; (**A**) The effects of $CaCl_2$ and $MgCl_2$ on proliferation of *cdc13-1* cells undergoing telomere dysfunction; (**B**) The effects of NaCl on proliferation of *cdc13-1* cells with additional genetic constructs used for experiments presented in Figure 1 and 4 and indicated on the left; (**C**) The effects of salt on proliferation of single polymerase subunit mutants.

Supplementary Figure 2: The effect of salt on proliferation of wild type and mutant cells treated with the following agents: (**A**) 0.01% MMS; (**B**) 120 mM HU; (**C**) 60 J/m2 UV; (**D**) 2.5mg/l Phleomycin; (**E**) mock treated.

Supplementary Figure 3: The effect of salt supplements on telomere length of *cdc1-1* cells incubated for 5 days in liquid YPD at the restrictive temperature of 27ºC. Southern blot shows telomere restriction fragments digested with Xho1 and probed with a labelled TG probe. The numbers 1-5 above the lanes indicate the days at of 27ºC. From left to right: molecular weight marker, wild type cells, *cdc13-1* cells at permissive temperature, *cdc13-1* cells incubated for 5 days on 20 mM NaCl (normal YPD); *cdc13-1* cells incubated for 5 days on 170 mM NaCl; *cdc13-1* cells incubated for 5 days on 450 mM NaCl. The arrow at the top of the day 5 (on 20 mM NaCl) indicates the amplification of Y'-subtelomeric regions, suggesting the emergence of Type I recombination-dependant survivors. The horizontal blue line indicates the wild type length of the terminal restriction fragments (e.g. the telomere plus about 1 kb from the Y' subtelomere). DNA loading is indicated below the Southern blot.

	A	B	C	D	E	F	G	H	I	J	K	L	M	N	O	P	Q	R	S	T	U	V	W	X	Y
**1**	6	2	3	2	5	2	4	1	6	2	2	2	4	2	4	1	6	2	3	2	4	1	2	1	3
**2**	3	1	1	2	2	2	3	1	1	2	2	2	1	1	1	2	2	2							
**4**	4	4	4	4	5	4	2	2	2	2	-	2													

Supplementary Table 1. Number of independent experiments related to Figures 1, 2 and 4. Rows indicate figure numbers (1,2 or 4). Columns indicate subpanels (A-Y) of specific figures. Numbers indicate how many independent experiments have been performed for each subpanel. For example, number 6 in the top left corner means that the experiment presented in Figure 1A (e.g. the quantification of ssDNA in subtelomeres during 480 min at 27ºC) has been performed 6 times. For experiments performed only once: either they reproduce previously published data (1), or show similar measurements at two different time points at the restrictive temperature.

1. Xue, Y., Rushton, M.D. and Maringele, L. (2011) A novel checkpoint and RPA inhibitory pathway regulated by Rif1. *PLoS Genet*, **7**, e1002417.

MIQE Real Time PCR data:	
Definition of experimental and control groups: Telomere dysfunction versus normal telomeres	E
Number within each group: 1 (control is time 0 versus later time points)	E
Assay carried out by investigator's lab? Yes	D
Acknowledgement of authors' contributions	D
Sample	
Description: Yeast cell cultures	E
Volume/mass of sample processed: 40 ml collected each time	D
Microdissection or macrodissection: None	E
Processing procedure: as described in the ChIP protocol	E
If frozen - how and how quickly? At -80ºC, within 1 min	E
If fixed - with what, how quickly? N/A	E
Sample storage conditions and duration: At -80ºC for a few days	E
Nucleic acid extraction	
Procedure and/or instrumentation: Glass beads, Centrifugation	E
Name of kit and details of any modifications: QIAquick PCR Purification kit (Qiagen)	E
Source of additional reagents used	D
Details of DNase or RNase treatment: N/A	E
Contamination assessment (DNA or RNA): N/A	E
Nucleic acid quantification: N/A	E
Instrument and method: Microcentrifuge; as described for the QIAquick PCR Purification kit	E
Purity (A260/A280): high	D
Yield: high	D
RNA integrity method/instrument:N/A	E
RIN/RQI or Cq of 3' and 5' transcripts: N/A	E
Electrophoresis traces	D
Inhibition testing (Cq dilutions, spike or other):N/A	E
Reverse transcription	
Complete reaction conditions: N/A	E
Amount of RNA and reaction volume: N/A	E
Priming oligonucleotide (if using GSP) and concentration: N/A	E
Reverse transcriptase and concentration: N/A	E
Temperature and time: N/A	E
Manufacturer of reagents and catalogue numbers: N/A	D
Cqs with and without RT: N/A	D*
Storage conditions of cDNA: N/A	D
qPCR target information	
If multiplex, efficiency and LOD of each assay: N/A	E
Sequence accession number: Yeast genes *PAC2, ERG26, YER188W* and *YER190W*	E
Location of amplicon: internal	D
Amplicon length: about 150 bp	E
In silico specificity screen: BLAST	E
Pseudogenes, retropseudogenes or other homologs? No	D
Sequence alignment	D
Secondary structure analysis of amplicon: N/A	D
Location of each primer by exon or intron: N/A (yeast)	E
What splice variants are targeted? N/A (yeast)	E
qPCR oligonucleotides	
Primer sequences:<br>**FOR *YER190W* aka Y'600 subtelomeres:** GAGATCAGCTTGCGCTGGGAGTTACC<br>CCGAAATGTTTTATTGCAGAACAGCCCTAT<br>**For *YER188W*:** AACGTACAGGTTACGATCGCGTCATTTTA<br>TCATGCCGTTCAAATTCTGAGGGTTCT	E

**For *PAC2*:** AATAACGAATTGAGCTATGACACCAA<br>AGCTTACTCATATCGATTTCATACGACTT	
RTPrimerDB identification number N/A	D
Probe sequences:<br>**FOR *YER190W* aka Y'600 subtelomeres:** ACAGGAATGCCGTCCAATGCGGCACTTTAGA<br>**For *PAC2*:** CTGCCGCGTTGGTCAAGCCTCAT<br>**For *YER188W*:** TAGCCGTTATCATCGGGCCCAAAACCGTATTCATTG	D**
Location and identity of any modifications: At 5'-3': VIC-TAMRA (PAC2 only) and FAM-TAMRA	E
Manufacturer of oligonucleotides: varies	D
Purification method: desalted	D
**qPCR protocol**	
Complete reaction conditions: Fast (1 hrs)	E
Reaction volume and amount of DNA: 25µl, amount of DNA varies between 0.01 and 30 ng/µl	E
Primer, (probe), Mg++ and dNTP concentrations: (final concentrations): 300 nM for primers, 200 nM for probes, Mg (as in 1xExTaq buffer), 0.2 mM dNTPs.	E
Polymerase identity and concentration: ExTaq (Takara)	E
Buffer/kit identity and manufacturer: ExTaq buffer (Takara)	E
Exact chemical constitution of the buffer	D
Additives (SYBR Green I, DMSO, etc.): None	E
Manufacturer of plates/tubes and catalog number: ABI	D
Complete thermocycling parameters: 1) 5 min at 95ºC; 2) 15 sec at 95ºC; 3) 1 min at 63ºC. 2) and 3) repeated 40 times	E
Reaction setup (manual/robotic): Manual	D
Manufacturer of qPCR instrument: Applied Biosystems	E
**qPCR validation**	
Evidence of optimisation (from gradients)	D
Specificity (gel, sequence, melt, or digest): Gel	E
For SYBR Green I, Cq of the NCT: N/A	E
Standard curves with slope and y-intercept: -3.2-(-3.6)	E
PCR efficiency calculated from slope: 88-104%	E
Confidence interval for PCR efficiency or standard error	D
r2 of standard curve: 0.98-1	E
Linear dynamic range N/A	E
Cq variation at lower limit: N/A	E
Confidence intervals throughout range	D
Evidence for limit of detection: as little as 0.01 ng/µl DNA detected	E
If multiplex, efficiency and LOD of each assay N/A	E
**Data analysis**	
qPCR analysis program (source, version): AB StepOne v2.0	E
Cq method determination: Standard, automated	E
Outlier identification and disposition: N/A	E
Results of NTCs: undetermined	E
Justification of number and choice of reference genes: *PAC2* and *ERG26* are centromeric (e.g. far from telomeres) and not directly affected by telomere dysfunction	E
Description of normalisation method: 10 µl of DNA standards at 2, 0.2, 0.02 ng/µl	E
Number and concordance of biological replicates	D
Number and stage (RT or qPCR) of technical replicates: 3	E
Repeatability: high	E
Reproducibility (inter-assay variation, %CV)	D
Power analysis	D
Statistical methods for result significance: Standard deviation	E
Software (source, version): AB StepOne 2.0	E
Cq or raw data submission using RDML	
